# Voices from Service Providers Who Supported Young Caregivers throughout the COVID-19 Pandemic in the Canadian Context

**DOI:** 10.3390/ijerph20156446

**Published:** 2023-07-26

**Authors:** Kristine Newman, Heather Chalmers, Sarah Ciotti, Arthur Ze Yu Wang, Luxmhina Luxmykanthan

**Affiliations:** 1 Daphne Cockwell School of Nursing, Toronto Metropolitan University, 288 Church Street, Toronto, ON M5B 1Z5, Canada; sciotti@torontomu.ca (S.C.); ze.wang@torontomu.ca (A.Z.Y.W.); luxmhina@torontomu.ca (L.L.); 2 Department of Child and Youth Studies, Brock University, 1812 Sir Isaac Brock Way, Saint Catharines, ON L2S 3A1, Canada; hchalmers@brocku.ca

**Keywords:** young caregivers, pandemic, service providers, qualitative, social determinants of health

## Abstract

This empirical research is part of a larger project beginning in 2020 and ongoing until 2023, exploring the impact of the COVID-19 pandemic on young caregivers aged 5–25 years and their families in Canada. This qualitative research utilizes the social determinants of health as a conceptual framework and a collective case study design to emphasize the voices and experiences of service providers (professionals offering services to young caregiver clients) during the COVID-19 pandemic, and exploring their perspectives on the impact of the pandemic on young caregivers and their families. The central research question guiding this study was “How do service providers (professionals) working with young caregiver clients in Canada describe the impacts of the pandemic on themselves, their professional praxis, and on their young caregiver clients?” The aim of this study was to develop a deeper understanding of the impact of the pandemic on young caregivers in Canada, from the perspectives of service providers, as well as to understand the experiences of service providers in their own voices. Data were collected from service providers working within three (3) different organizations offering programs and services to young caregiver clients in Ontario, Canada. In total, six (6) individual interviews were conducted with service providers who were directors/program managers, and four (4) group interviews were conducted with thirteen (13) service providers who were frontline staff members who worked directly with young caregivers and their families. In total, nineteen (*n* = 19) unique service providers participated in this study. Our findings point to two primary overarching themes, namely (1) service providers’ responses to the pandemic and (2) observations by service providers about the impacts of the pandemic on young caregivers, and a secondary theme, (3) positive outcomes from the COVID-19 pandemic on young caregivers, that emerged through the analysis. The pandemic led to increased demands for services by young caregiver clients. Service providers were required to adapt their service delivery methods in order to comply with public health guidelines. They shared how their work impacted their mental health as they struggled to maintain personal and professional boundaries while working from home during the pandemic. Importantly, service providers identified similar, simultaneous, and co-occurring impacts of the pandemic between their young caregiver clients, including isolation, difficulties in navigating online spaces, and challenges in navigating boundaries while working from home with family members.

## 1. Introduction

A young caregiver is a person, under the age of 25 years, who provides care to another individual (often family members or friends) who lives with an illness, disability, injury, and/or challenge to activities of daily living. Without appropriate and adequate support, resources, and services, young caregivers can experience the “young carer penalty” [1] which describes short- and long-term harms to their physical, mental, and social health and wellbeing as a result of a limited capacity to fully engage in the activities required for their academic, personal, social, and professional development [2,3]. Available data indicate that young caregivers in Canada spend 14–27 h per week caring for others—equivalent to a part-time job—and that their unpaid work contributes CAD 25,000 to 50,000 in annual savings for the family and healthcare system [1,2]. Despite their contributions, young caregivers represent a marginalized and under-served group in Canada; there are few resources and services available nationally to support young caregivers and their families, and the majority of Canadians remain uninformed of the challenges faced by this population.

Very few formal programs are currently available for young caregivers in Canada: in 2015, which is the last time that existing programs in Canada for young caregivers were documented, there were only three [4], and, based on information gathered by our community partners, there are now 10 as of 2021. The existing services are simply inadequate: an estimated 1.25 million Canadian caregivers are aged 15–24, and less than 1% of them are being supported through programming specifically designed for them [1]. In contrast, the UK has recognized and actively supported young caregivers for more than 25 years, and, in 2015, they had over 350 programs available that were specifically tailored to young caregivers [5]. Young caregivers in Australia have access to programs and even some financial support for postsecondary education [6], and, in the UK, young caregivers also have distinct legal rights, and access to services and policies designed to support them have been integrated into local schools, social services, and healthcare systems [7]. In Canada, the most commonly accessed federal welfare programs for caregivers, such as the Canada Caregiver Credit as well as Employment Insurance Caregiving Benefits and Leave, are only intended for working adults, and Canada in general lacks the necessary policies to support young caregivers financially who cannot work as a result of being both a caregiver and student [8]. Currently, no national legislation or action plan mentions young caregivers. 

Research on the lived experiences of young caregivers and their families in the Canadian context is growing [1,3,4,6,9,10,11]; however, the available research does not report on the impact of the COVID-19 pandemic on young caregivers and their families in Canada. Even the study conducted by Newman and others (2022) [10], which collected data on young caregivers during the pandemic living in Ontario, Canada, did not capture the impact of the pandemic on young caregivers. The study collected data on young caregivers living in rural versus urban communities in phase 1 of their study (conducted before 2020) and, in phase 2 (conducted in 2020), evaluative data on an emergency planning video that was developed to help young caregivers and their families as the pandemic progressed.

Emerging in 2019, COVID-19 became a globally significant public health threat officially in the spring of 2020 [12]. Although young people (without underlying health conditions) were at a low risk of serious and adverse health outcomes from the disease in 2020 [13,14], research has highlighted how pandemic restrictions (in the form of lockdowns and social distancing) led to adverse psychological and mental health effects for young people [15,16,17,18,19,20]. For instance, internationally, schools were forced to close [21], which led to increased social isolation [22] for children, adolescents, and emerging adults. A study by Schoon and Henseke (2022) [23] suggests that young people aged 16–25 experienced adverse mental health effects from the pandemic at a disproportionate rate based on the pre-pandemic conditions in which they were living. Thus, as young caregivers already experience significant adversity, it can be argued that the pandemic exacerbated the pre-existing inequities experienced by this population. A systematic review by Lacey, Xue, and McMunn (2022) [24] looked at articles published on the mental and physical health of young caregivers before 23 January 2022, and their search revealed no articles published in the context of the pandemic on young caregivers. Our search for peer-reviewed literature on the impact of the pandemic on young caregivers revealed two articles. One looked at the impact of social distancing on young caregivers in the UK [25] and the other looked at the Italian context, exploring the impact of parental illness and other ill family members on COVID-19-related and general mental health outcomes [26]. Blake-Holmes and McGowan (2022) [25] reported that young caregivers experienced an increase in the care that they were required to provide due to a reduction in external formal support/services as they became unavailable or inaccessible. Social distancing measures placed a significant amount of stress on young caregivers that impacted many areas of their lives, including the ability to take care of their mental health, the efficacy of coping strategies, and managing school/work from home. Landi and others (2022) [26] collected data from 1823 Italians aged 18–29 via an online survey (1458 reported no ill family members and formed the non-carer group) and found that young adult carers in Italy reported poorer mental health across all outcomes compared to non-carers, which was an expected finding.

A comprehensive review of the existing literature focused on service providers for young caregiver clients during the pandemic was performed but yielded limited results. For example, a search on PubMed with the criteria “(young AND caregiver OR carer) AND (COVID OR pandemic OR 202* OR lockdown OR restrictions OR corona) AND (organizations OR service OR healthcare OR work* OR staff OR volunteer)” identified fifty-four (54) papers that potentially included service providers’ perspectives. Unfortunately, none of the papers offered insight from service providers that supported young caregivers in the context of the pandemic. Based on this discovery, the research team wondered if any of the articles at least offered any insight into the Canadian context. Of the 54 papers, only one of them was published based on the Canadian context, and it was an article that we were familiar with that was published by Stamatopoulus in 2016 [4]. This is likely due to the limited availability of young caregiver organizations across the country, as we noted previously. This study addresses a gap in the current body of scholarship focused on young caregivers and service providers who support young caregivers in the Canadian context and also addresses a gap in the international literature on service providers who supported young caregivers within the context of the COVID-19 pandemic.

### 1.1. Conceptual Framework

Building upon the available international literature focused on organizations working with young caregiver clients and their families [4,27,28,29], this study utilizes the social determinants of health [30] as a conceptual framework, to explore the perspectives of service providers (professionals) working in non-profit organizations offering services and support for young caregivers and their families in Canada during the pandemic. The World Health Organization (2008) [30] defines the social determinants of health as “the non-medical factors that influence health outcomes. They are the conditions in which people are born, grow, work, live, and age, and the wider set of forces and systems shaping the conditions of daily life. These forces and systems include economic policies and systems, development agendas, social norms, social policies and political systems” (para. 1). For young caregivers, these conditions often centre on their caregiving responsibilities. This conceptual framework offers a perspective from which to analyze the social inequities experienced by young caregiver populations. Young caregivers in Canada are a marginalized population that is underrepresented in the academic literature.

### 1.2. Research Aim

This empirical research was part of a larger project beginning in 2020 and ongoing until 2023, exploring the impact of the COVID-19 pandemic on young caregivers aged 5–25 years and their families in Canada. The central research question guiding this study was “How do service providers (professionals) working with young caregiver clients in Canada describe the impacts of the pandemic on themselves, their professional praxis, and on their young caregiver clients?” Service providers working with young caregivers and their families provide an important entry point into formal systems for young caregiver clients. These professionals play an important role in offering support and resources, with the goal of increasing public awareness and funding for young caregivers and their families while offering direct service and support. The aim of this study was to develop a deeper understanding of the impact of the pandemic on young caregivers in Canada, from the perspectives of service providers, as well as to understand the experiences of service providers in their own voices. This research offers additional insights into the experiences of young caregivers and their families and offers a relevant point of comparison with research with young caregiver clients themselves. This research contributes additional information that can inform social policy in this area.

## 2. Materials and Methods

**Figure 1:**
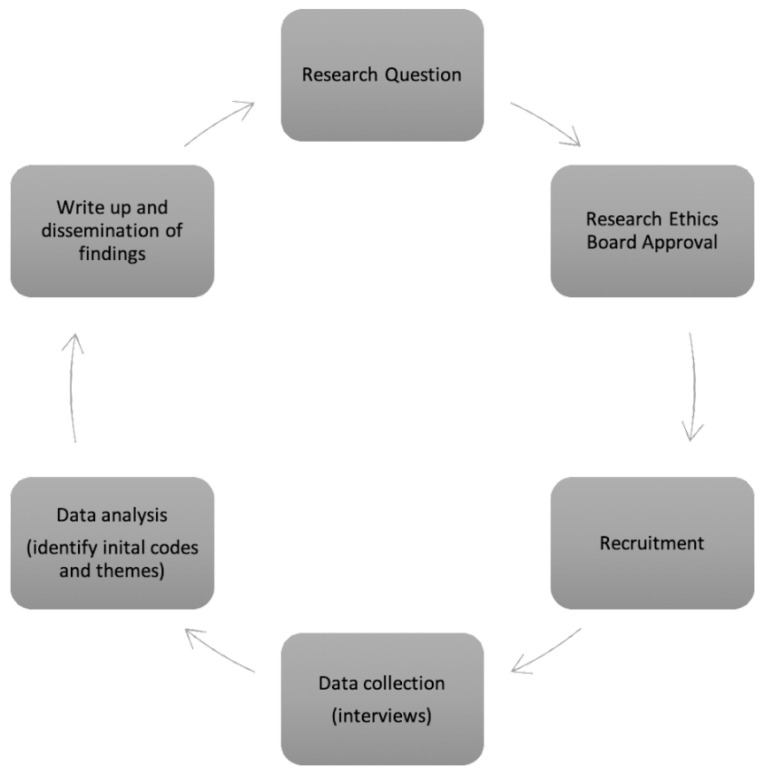
Methods and procedures used in this study.

The case study methodology [31,32,33,34] informed the materials and methods used in this study to analyze the impacts of the COVID-19 pandemic on professionals working with young caregivers, including their assessments of the impact of the pandemic on young caregivers themselves. Well established in small-scale social studies [33], a case study allows for an in-depth analysis of complex phenomena within a natural setting [34] and is bounded in time and place [32]. This empirical research utilized a collective case study design [32] and included data collected from professionals working with young caregiver populations in various young caregiver organizations in Ontario, Canada, to gain different perspectives on the experiences of Canadian service providers working with young caregivers during the COVID-19 pandemic. The methods for data collection included semi-structured individual and group interviews (please see Supplementary Materials for semi-structured interview questions for directors/managers and service providers who support young caregivers directly) with service providers from non-profit organizations working with young caregivers in Ontario, Canada. Interview questions were focused on the impact of the pandemic on their organizations, their work, and their clients (young caregivers). Figure 1 outlines the methods and procedures used in this study.

These methods were chosen by the research team as they allowed for a deeper understanding of the experiences of service providers working with young caregivers in Canada during the pandemic in their own voices, thus addressing the aim of the study.

### 2.1. Setting

Data were collected from three (3) different settings (organizations offering programs and services to young caregiver clients) in Ontario, Canada. Nineteen (*n* = 19) service providers participated in total. Six (6) individual interviews were conducted with directors/program managers and four (4) group interviews were conducted with thirteen (13) staff members who worked directly with young caregivers and their families. The semi-structured group and individual interviews started in January of 2022. We originally intended to start in 2021 (a year into the pandemic), but, through our observations and conversations with our community partners, it was apparent that they were still in a time of constant policy change due to public health restrictions and staff would find it challenging to speak with us. Thus, to mitigate the risk of causing participants to feel emotionally or mentally vulnerable, we delayed the research until 2022, when the situation was relatively more stable, and gave service providers some time to reflect on the challenges, experiences, and successes that they and their clients experienced. Directors/program managers were not included in the group interviews, based on the understanding that the participants would find some topics (i.e., burnout, social isolation, impact of constant changing policy changes and procedures) easier to discuss without their supervisors in the group. Similarly, issues such as resource cuts, liquidity issues, and difficult decisions that were made, if they occurred, would have been difficult to discuss with one’s employees present. Ethics clearance was obtained from Toronto Metropolitan University’s Research Ethics Board (formerly Ryerson University Research Ethics Board) on 8 July 2021 (No. 2021-216-1) and Brock University’s Research Ethics Board on 22 July 2021 (No. 21-014).

### 2.2. Sampling and Recruitment

Participants were recruited via selective sampling. The research team engaged with community partners who provided services/support/resources to young caregivers and their families, i.e., the Young Caregivers Association (served all of Ontario during the pandemic), the Ontario Caregiver Organization (served all of Ontario during the pandemic), and the Young Carer Program (mainly served Toronto during the pandemic), to recruit service providers working with young caregivers during the pandemic. Participants were invited to individual and group interviews focused on their work with young caregivers.

Interested participants were screened via email to determine their eligibility for the study and to determine whether they were a manager/director or a service provider who worked directly with young caregivers during 2020 and/or 2021. Managers/directors were provided with a consent form to review for an individual interview, and service providers were provided with a consent form for group interviews. Consent was completed and collected online via Google Forms. During the process of completing the online consent form, participants were also asked to create a participant code to be used as their display name during the Zoom interview. Participants were then provided with a Doodle poll link for them to anonymously indicate their availability, to allow the research team to identify the best time or times to schedule group interviews. All interviews were conducted over Zoom by a graduate-level research assistant who had had over eight (8) years of field experience in qualitative data collection and five (5) years of experience in supporting research work on young caregivers. Participants were asked to be in a space that ensured audio and visual privacy. The interviews were audio-recorded only for the purpose of transcription and participants had the option of having their cameras turned on or off. Group interviews lasted 1 h and 30 min and individual interviews lasted between 45 min and 1 h. The first and second group interviews both consisted of three (3) staff members who worked directly with young caregivers and were both held on 11 January 2022. The third group interview was held on 20 January 2022, consisting of three (3) participants, and the fourth was held on 25 January 2022 with four (4) participants. Four (4) individual interviews were held with directors and two interviews were held with managers during February of 2022.

### 2.3. Participants

Participants were required to be service providers who worked directly with young caregivers on a regular basis as part of their roles and responsibilities during COVID-19 and/or through the recovery/reopening/lifting of restrictions. The following were used as inclusion criteria for service providers: (1) older than 18 years of age; (2) provided support, resources, and/or services to a young caregiver as part of their responsibilities in a formal role during/after 2020; (3) lived in Ontario, Canada; (4) could speak English.

Of the 13 participants that supported young caregivers directly, eight (8) were employed as full-time staff and the other five (5) were committed volunteers. Of the eight (8) employed full-time staff, four (4) had backgrounds in early childhood education and/or child and youth care, with three (3) to seven (7) years of experience in working with young caregivers. The other half had an educational background in social work and five (5) to seven (7) years in supporting young caregivers. Of the five (5) participants who were volunteers, two (2) had been in their roles for two to three years and were students in child and youth studies. Two (2) committed to being volunteers to contribute both their backgrounds in mental health and insights from their lived experience as young caregivers with four (4) years of experience in their roles supporting other young caregivers. One (1) was an adult caregiver who had experience in working with youth and had supported young caregivers for two (2) years.

Of the six (6) directors and managers that were individually interviewed, five (5) of them had five (5) to eight (8) years of experience contributing to work in support of young caregivers. One (1) manager was relatively new to the role and had one year of experience working in her role of managing young caregiver services. Directors and managers had a variety of different education backgrounds, including teaching, early childhood education, child and youth studies, and social work. Prior work experience before beginning their roles that contributed to providing services for young caregivers included teaching, family counselling for caregivers, social work, and child and youth work.

Participants identified three (3) main roles, i.e., clinical support, service delivery, and facilitation and advocacy, in their work with young caregivers during the pandemic. Table 1 outlines these three main roles.

### 2.4. Data Analysis

Findings were systematically reviewed multiple times and analyzed by the research team using reflexive thematic analysis [35]. Once the interviews were completed, the audio recordings were transcribed verbatim, the data were removed several times by the research team (coding was done by a young caregiver aged 24 who had been providing care since she was 8, a PhD candidate in child/youth studies who was a Registered Psychotherapist at the time of this publication, and a graduate student studying law/public health who had been involved with research on young caregivers for five years) and initial codes were generated, and initial themes were identified through reflexive thematic analysis [35]. An inductive analytical approach was used, and the primary and secondary themes were decided through collaborative discussions between the research team members.

## 3. Results

The findings pointed to two primary overarching themes, namely (1) service providers’ responses to the pandemic and (2) observations by service providers about the impacts of the pandemic on young caregivers, and a secondary theme, (3) positive impacts of the COVID-19 pandemic on young caregivers, that emerged through the analysis.

### 3.1. Service Providers’ Responses to the Pandemic

Service providers overwhelmingly described increases in client volume and client engagement. The increase in demand for services during the pandemic resulted in increased access to online clinical services that existed previously, so staff/volunteers that assisted/facilitated in-person programming had to shift to providing online programming to meet the demand. Online services were not as frequently used prior to the pandemic, but with the growth in the popularity of applications such as Zoom during lockdowns, this led to the expansion of service delivery provincially (i.e., in Ontario) and nationally (i.e., across Canada) given that more people were turning to virtual formats as in-person programs moved online or were stopped due to public health restrictions. As reported by one participant, “There was just a huge increase in the amount of use of online recreation services, but also clinical services so I’ve had more youth become aware of help lines in our regions and across the province than ever before” (counselor and program developer/facilitator for young caregivers; employed full-time; provided counseling to young caregivers and families of young caregivers and developed/facilitated recreational programs for young caregivers; had a background in social work). Service providers were required to adapt their service delivery models to meet the needs of clients while ensuring adherence to public health guidelines. As a result, service providers identified changes in their roles, including offering telephone “check-in” meetings with clients, delivering food, helping families to access government benefits (e.g., Canada Emergency Response Benefit [CERB]) provided financial support of up to CAD 500 every week to employed and self-employed Canadians who experienced a loss of income because of COVID-19), and other financial support, making videos for young clients (e.g., videos about breathing techniques), and making (and delivering) “at-home kits” with equipment for programs. Some service providers noted that they continued offering in-person services to families that desperately needed them in person.

As reported by one participant,

*“Yeah in 2019, we would have been physically in the office, feels like a very long time ago now. We would have been in the office and seeing people every day and doing more like in-person check-ins by going to families’ homes, having staff meetings altogether, facilitating in-person programming with like our programs and partnership in schools and things like that. And then in 2020, we were fully virtual so no one was going into the office at all, we had to switch to Zoom for Health Care, we had to switch what we were able to actually offer our young caregivers and families. In the beginning of the pandemic, we did little like challenges, like just through text messages to kind of keep them engaged and to help them stay connected with us as we made our big switch to Zoom. And then pretty much right away, we were able to switch to doing like Zoom groups and delivering kits to people homes for activities and programming and stuff like that which followed social distancing measures”* (director of programs and services; employed full-time; oversaw the day-to-day operations of, and developed policies and procedures for, the development/facilitation of services/programs for young caregivers and their families; had a background in child and youth studies).

Importantly, service providers noted that they were required to adapt their responses to meet more diverse client needs due to clients seeking their services from further away, since there was no distance restriction that in-person programs once posed to families, which resulted in accelerated changes to previous service delivery models. As noted by one participant, their client load doubled and tripled “from 6 clients to having 12 to 18 clients a month due to increased need for support during COVID-19 for caregivers and their families” (counselor and program developer/facilitator for young caregivers older than 12 years old; employed full-time; provided counseling to young caregivers and families of young caregivers over the age of 12; had a background in social work). At the same time that service providers were attempting to manage their increased caseloads and adjust to running programs virtually, they also had to navigate new issues/family dynamics with young caregivers and their parents caused by public health restrictions (e.g., virtual schooling, gaming/screen time): “OMG, yeah, it was such a dichotomy between the parents and the kids because we support the parents and the kids. And it was really hard on our end to try to explain to parents like, yeah, your child’s online a lot right now, but they have no other way to talk to any of their friends or anyone, that is their age, and now they’re able to have fun for a couple hours a day” (counselor and program developer/facilitator for young caregivers; employed full-time; provided counseling to young caregivers and families of young caregivers and developed/facilitated recreational programs for young caregivers; had a background in social work).

In some cases, service providers noted the changes to their work and workload, requiring them to receive additional training (e.g., work-related training, government training, health network training). This training helped service providers to better provide clinical support virtually to young caregivers and their families. Service providers identified newly acquired skill sets, which allowed them to facilitate more opportunities for young caregivers to engage in programs, groups, and clinical options:

*“I’m registered to the Ontario College of Social Workers and that’s where I got a lot of my support and resources in connecting with various different therapists. And I was lucky, I was reflecting on my trainings last year, and it was the most trainings that I’ve ever taken compared to when I started my enrolment with this organization. So it was really kind of good to see that, too, as well, but what was helpful was a lot of this trainings were offered at no cost, so I felt very supported with my college and them being able to provide this great training from COVID-19 to discussions to different modalities on ways that we could support our clients and gave me the tools to feel prepared and empowered. You know whether it was virtual challenges, how do you kind of go around having virtual groups, how do you provide engagement? And also tapping into different therapists who are taking those trainings and then seeing what was working in theirs. I would find myself writing down tips to see what could also be replicated in my own kind of work”* (licensed social worker; employed full-time; provided social work services to young caregivers and their families, such as providing information and guidance on resources, benefits, and services that young caregivers and their families could access; background in social work).

Service providers identified that they were required to adapt their approaches with an emphasis on understanding and accommodating the varying needs of diverse young caregiver clients. These changes included building referral lists to outside agencies and developing community connections, prioritizing services to traditionally under-represented and social excluded communities (e.g., pride group, Indigenous services, and support for Black, Indigenous, and people of colour (or BIPOC) communities), branching out to support schools, and expanding services for young caregivers aged 5–25 years (from 5–12 years). As reported by one participant, “There’s a lot of assumption that happens in the community around ‘oh we’ll make a webinar’, ‘we’ll make a support group’ or we’ll do this because that’s what’s been done in other spaces, but we live in such a diverse environment with such a range of people with different needs and it’s so important to be able to represent the needs and requests of this population. We tried that as an organization with our website and we brought youth to the table and asked them what they needed” (online support group and outreach facilitator; volunteer; facilitated and scheduled support groups with adult and/or young caregivers and reached out to different organizations within the province to build awareness of and engage caregivers; had lived experience as a caregiver). Additionally, organizations shortened program durations to allow more people to access their programs and to offer more types of programs for young caregivers of different ages. Organizations implemented new types of programs to keep caregivers engaged. Examples included “tabletop therapy group where board games are played” (counselor and program developer/facilitator for young caregivers older than 12 years old; employed full-time; provided counseling to young caregivers and families of young caregivers over the age of 12 and developed/facilitated recreational programs for young caregivers over the age of 12; had a background in social work) and “‘text message challenges’ for kids who were tired of Zoom or mini scavenger hunts where kids would take pictures and send them back” (counselor and program developer/facilitator for young caregivers between 5 and 12 years old; employed full-time; provided counseling to young caregivers and families of young caregivers between the ages of 5 and 12 and developed/facilitated recreational programs for young caregivers between 5 and 12 years old; had a background in social work).

Service providers described the impact of role change(s) during the pandemic on their mental health and the importance of connections with their colleagues: “It was the middle of a pandemic and the workers were going through their own adjustments, so quickly having to take on 7 new roles was not ideal because it resulted in burn out” (counselor and program developer/facilitator for young caregivers identified through partnerships with schools; employed full-time; worked with schools to develop programs for students who identified as young caregivers and also provided counseling services to them and their families; had a background in social work). Importantly, service providers acknowledged the challenges of working from home while maintaining work/life boundaries. As noted by a participant, “since [staff members] were doing work from home, there was no change in environment after a difficult/heavy day at work and the lack of boundaries affected their mental health” (counselor and program developer/facilitator for young caregivers between 5 and 12 years old; employed full-time; provided counseling to young caregivers and families of young caregivers between the ages of 5 and 12 and developed/facilitated recreational programs for young caregivers between 5 and 12 years old; had a background in social work). Finally, the pandemic also had an impact on service providers themselves. As one participant noted, “[my colleagues] were burning out, taking extended sick leaves, and taking time off for their own mental health” (online support group and outreach facilitator; volunteer; facilitated and scheduled support groups with adult and/or young caregivers and reached out to different organizations within the province to build awareness of and engage caregivers; had lived experience as a caregiver).

### 3.2. Observations by Service Providers about Impacts of the Pandemic on Young Caregivers

Service providers noted several barriers experienced by service users, including negative attitudes toward online platforms and services, uncertainty about ongoing pandemic disruptions (from lockdowns and reopening), overwhelming feelings from increased caregiver responsibilities, and a lack of breaks/respite for young caregivers and their families from COVID-19 prevention and management. This included several issues with online platforms and services for young caregiver clients and their families. Participants described how parents struggled with allowing young people too much access to technology as a substitute for outdoor recreational activities, which were no longer an option given the risk of community spread of COVID-19:

*“Discord, Roblox, Minecraft, and Messenger Kids are the four main ones I heard the young caregivers using during the pandemic to connect with their friends. I always tried to talk to parents. I have a son myself and he got really into video games over the pandemic too, because what else are you going to do? But what’s hard, I think, for our families, is that if we look at the kids and if they were outside playing with their friends for four hours, we would have absolutely no issue with it, but playing online with their friends for four hours was like ‘that’s too long,’ but in the pandemic, there is no playing outside, so it was a big struggle and I think that’s one of the impacts that I saw in kids there was a lot more fighting between parents and kids. There was a lot more of not seeing eye-to-eye because these kids grew up some of them for half their life in the pandemic now because we support five and up so some of them were like two and a half, when the pandemic started and now they’re five so they’re at an age point where most of their life has been online and accessing online services and accessing online games and that’s how they communicate”* (counselor and program developer/facilitator for young caregivers; employed full-time; provided counseling to young caregivers and families of young caregivers and developed/facilitated recreational programs for young caregivers; had a background in social work).

Participants also reported that their program attendees became “zoomed out” after spending all day on the computer in school and felt a lack of connection due to the difficulty of building a rapport online, compared to in person. Additionally, young caregiver clients experienced challenges with privacy and Wi-Fi access while accessing services and support remotely: “something that was a huge problem for some of the young carers, which I can relate to, living in small apartments with a lot of people made participation so difficult … there are some people where their home is just not a suitable environment because of Wi-Fi definitely, just their families in the background, no privacy, having their siblings and parents overhear the conversations, even if they’re not trying to. That was definitely an issue” (program assistant/facilitator; employed full-time; assisted with and/or facilitated existing recreational programs developed for young caregivers; background in early childhood education). As noted by another participant, “Clients were used to it in-person and transitioning to virtual involved barriers such as access to the internet and computers. It is hard to share personal and traumatic experiences virtually because of a lack of full human connection” (licensed social worker; employed full-time; provided social work services to young caregivers and their families, such as providing information and guidance on resources, benefits, and services that young caregivers and their families could access; background in social work).

According to service providers, young caregiver clients expressed caution and confusion around facilities opening up due to the risk of closing again, including schools, a lack of extracurricular activities, and programs/services shutting down (including healthcare) or being more difficult to access virtually (e.g., therapy). One participant stated, “entering school online and then suddenly in class while having to wear masks and then switched to online again, adjustment and lack of stability was difficult for many students” (counselor and program developer/facilitator for life skills; provided counseling services to young caregivers and their families as well as developing and facilitating programming that taught young caregivers life skills such as, but not limited to, studying effectively, cooking, saving money, preparing for job interviews, and online safety; background in social work). As with many people during the pandemic, young caregivers and their family members experienced challenges with a loss of employment, underemployment, and/or precarious employment. Participants reported that 2021 was significantly more challenging, financially, for families compared to 2020, because of the end of CERB in December of 2020. As expressed by one participant,

*“So, when CERB ended at the end of 2020, I know a lot of our families have someone at home with a chronic health issue, so they still couldn’t return to work because there was concerned about the health and safety of their family member, so now they’re losing the financial support from CERB, but they also can’t go back to work full time. Or, they still have a child at home still and they can’t afford childcare because childcare services weren’t fully open yet or their child has immune issues. So that was in that regard, I would say it kind of got worse, at one point and a lot more financial strain happened on families, to the point, just as an example, one of my younger clients who is now 13 but 12 at the time, started making and selling things on Facebook marketplace, to try to make money to help their family, without their family knowing because they were so concerned about their family’s financial situation because they couldn’t return to work because of the family situation”* (counselor and program developer/facilitator for life skills; provided counseling services to young caregivers and their families as well as developing and facilitating programming that taught young caregivers life skills such as, but not limited to, studying effectively, cooking, saving money, preparing for job interviews, and online safety; background in social work).

Service providers identified their clients feeling overwhelmed from increasing caregiver responsibilities and a lack of breaks/respite, which presented significant challenges for young caregiver clients and their families. Young caregivers had to stop participating in the program to attend to their caregiver roles because it was conducted from home. They experienced challenges with motivation, self care, coping, and lost patience with the pandemic: “caregivers no longer had an escape from their roles because they had no change in environment for things such as school or work” (online support group and outreach facilitator; volunteer; facilitated and scheduled support groups with adult and/or young caregivers and reached out to different organizations within the province to build awareness of and engage caregivers; had lived experience as a caregiver). Some young caregivers had loved ones who passed away during the pandemic, and restrictions added layers of difficulty to activities such as funeral planning, wills, and estates. Families were overwhelmed, and caregiving responsibilities felt like they had increased: “The urge to get back to normalcy that affected their mental health and behaviours such as being overwhelmed and a lot of caregivers, even as young as six years old, noticed that programs ran shorter and were reflecting the worry and fear of their parents” (counselor and program developer/facilitator for young caregivers between 5 and 12 years old; employed full-time; provided counseling to young caregivers and families of young caregivers between the ages of 5 and 12 and developed/facilitated recreational programs for young caregivers between 5 and 12 years old; had a background in social work).

Service providers identified that a greater fear of COVID-19 was prevalent in caregivers, especially for families who took care of persons who were immunocompromised. As a result, COVID-19 prevention strategies were implemented as per public health guidelines aimed at stopping the spread, but there were still significant psychological challenges for their clients who cared for someone who was immunocompromised:

*“That fear that was talked about, like getting other people sick, that really impacted our families. A lot of our families do have immunocompromised people in their family so suddenly they were really uncomfortable with receiving help and they were having to choose between putting their family at risk and accessing resources that were incredibly valuable to them. Even when we started doing one to ones again it was that hit, like okay, we are doing one to ones, but there’s always an inherent risk and families were just at one point just so desperate that they were willing to accept that risk because it was between that and mental health, and I think it just put families in a situation where they had to prioritize stuff they weren’t ready to prioritize and wage on risks that they weren’t really prepared to take”* (counselor and program developer/facilitator for young caregivers identified through partnerships with schools; employed full-time; worked with schools to develop programs for students who identified as young caregivers and also provided counseling services to them and their families; had a background in social work).

Additionally, some young caregivers and their families struggled with public health guidelines; families were divided about government policies, which made it difficult for service providers to bridge these conversations; families struggled with understanding who was eligible for vaccination as a caregiver, and young caregivers who became sick and had to isolate themselves from their families were unable to provide care. One participant stated that “a divide was formed in some families and society about things such as vaccines which made workers feel helpless on how to approach it” (licensed social worker; employed full-time; provided social work services to young caregivers and their families, such as providing information and guidance on resources, benefits, and services that young caregivers and their families could access; background in social work). Another participant noted, “I had to have discussions with youth about advocacy and how to get the vaccine when you were under the age of 55 because a lot of caregivers didn’t have the confidence to say that they are caregivers and need to be fully vaccinated because they live with someone who is immunocompromised” (online support group and outreach facilitator; volunteer; facilitated and scheduled support groups with adult and/or young caregivers and reached out to different organizations within the province to build awareness of and engage caregivers; had lived experience as a caregiver).

Participants identified that their young caregiver clients needed physical resources such as headphones (enabling more privacy by helping to make young caregiver clients more comfortable talking about issues when family could hear them), reliable access to the internet (particularly for clients in rural areas, where internet connections were especially poor and uploading a video took hours), and internet-enabled devices (e.g., tablets, laptops), which were important for young caregivers during the pandemic. Young caregivers and their families often relied on access to the internet to participate in remote school, programs, and services, creating disparities for those who had versus did not have as much access to technology. As noted by one participant,

*“I just think that there are younger people than people might think, who either don’t have access financially like someone who might be between apartments, they’ve lost their job, what have you or they don’t have or maybe they only have access to like a smartphone like they don’t or a tablet or they don’t have a computer they don’t have good Internet access, they live rural or remotely. And just recognizing that online isn’t like a be all end all solution and having some other option like phone is a really good option for some people it’s accessible, it’s cheapish because libraries are the other place that we would recommend if you need Internet access to go. They were all closed by the pandemic, same with universities, which often had computer labs that were not available so support that young people might have relied”* (program lead for young caregivers and peer mentoring program; employed full-time; oversaw the planning, delivery, growth, and evaluation of peer-based support resources and programs for adult and young caregivers; background in psychology and peer support).

Another participant noted similar challenges with access to technology and privacy (i.e., not everyone had enough private rooms/spaces for their family members) and looked for grants/support to facilitate access to technology, to no avail:

*“And a lot of our kids didn’t have access to headphones and their family couldn’t afford to get them a headphone with a microphone, so they weren’t able or weren’t comfortable talking to me about their issues because they were like ‘my family will hear me.’ And that was a really big barrier and it would have made my job, a lot easier if we could just give those things to them. And we looked into it, but there was nothing. If we could have found some type of like grant or support for organizations to help get their clients things like headphones or a webcam for some kids who didn’t have a webcam and I couldn’t see them. And they just wanted to be included in the conversation, but they were a name the whole time and it felt weird talking when they were just a name and things like that. Like little tech things that can add up really quickly if you’re buying two or three hundred of them. Because it really was a barrier for a lot of our families like they just didn’t feel safe talking in our programs about certain things because they didn’t have small technology pieces like headphones. Like I don’t know if you guys found that in the program, but I was struggling hard and clinically with finding spaces in people’s homes where they felt safe, yeah, that was difficult”* (counselor and program developer/facilitator for young caregivers older than 12 years old; employed full-time; provided counseling to young caregivers and families of young caregivers over the age of 12 and developed/facilitated recreational programs for young caregivers over the age of 12; had a background in social work).

These resources required an investment in funding to offset the financial burden on young caregivers and their families. As expressed by another participant, they believed that it would be helpful to have a type of support that could be created for young caregivers: “caregivers are incurring a significant cost out-of-pocket even though healthcare is free and it is hard to get a loan as a young caregiver, thus things such as grants to pay for PSWs [personal support workers] would have a huge impact” (program lead for young caregivers and peer mentoring program; employed full-time; oversaw the planning, delivery, growth, and evaluation of peer-based support resources and programs for adult and young caregivers; background in psychology and peer support).

### 3.3. Positive Outcomes of the COVID-19 Pandemic for Young Caregivers

A secondary theme, positive outcomes of the COVID-19, emerged through the data analysis. Specifically, five service providers described positive impacts of the COVID-19 pandemic on young caregivers and their families. For example, participants noted that their young caregiver clients reported experiencing a reduction in bullying during remote learning, which allowed them to better focus on their studies. Participants also indicated that some young caregiver clients benefitted from independent learning, skill development, personal growth, and the freedom to explore self-concepts (e.g., gender, romantic orientation), which allowed more authentic growth without the peer pressure experienced in school:

*“I know a lot of the kids I work with who were being bullied suddenly the bullies weren’t there anymore, and they were able to better focus on their studies. And some kids, their learning style is much more independent and this independent learning style over the computer was so good for them, because they’re like I can actually learn the way I want to learn and get way more out of it. And, as well as kids kind of like getting to find out who they are, without the pressure of their peers. We had a lot of kids who, if you look at the beginning of the pandemic like right before, versus now, they look entirely different; like their looks are different, their hair is different, what the music they like is all different. But it is all more authentic to who they are, and it’s less like ‘I’m going to try to fit in with my classmates.’ Because they’re not seeing them, it’s just them now, and they were like ‘I can actually explore who I am and what I want’ and we’ve had like a lot of youth like come out during the pandemic in regards like their romantic/sexual orientation and gender journeys because they’ve had more time without the pressure of everyone else to really look at who they are”* (counselor and program developer/facilitator for young caregivers older than 12 years old; employed full-time; provided counseling to young caregivers and families of young caregivers over the age of 12 and developed/facilitated recreational programs for young caregivers over the age of 12; had a background in social work).

Online schooling and working from home allowed for more flexibility and options when accessing support for some young caregivers. As one participant noted, “teens that were too shy to come to program for reasons such as fear of not being accepted were able to come to program without their videos/cameras on without judgement” (counselor and program developer/facilitator for young caregivers; employed full-time; provided counseling to young caregivers and families of young caregivers and developed/facilitated recreational programs for young caregivers; had a background in social work). For some families, decreased access to structured activities reduced family stress and offered more opportunities for rest and relaxation. However, this did not last for everyone: “there was a ‘honeymoon phase’ at the beginning of COVID where things slowed down and households became less chaotic because there were less appointments and less people coming in and out of homes” (counselor and program developer/facilitator for young caregivers between 5 and 12 years old; employed full-time; provided counseling to young caregivers and families of young caregivers between the ages of 5 and 12 and developed/facilitated recreational programs for young caregivers between 5 and 12 years old; had a background in social work). Participants noted additional benefits to service providers in terms of knowledge generation and mobilization. Service providers identified that they were still able to build a rapport with young caregivers and families virtually, and some programs were planned to continue in this format to better serve clients. As expressed by one participant,

*“Balancing school… a lot of the youth that I supported were full time caregivers, going to school full time and often were also working part-time or full-time at the same time, so one of the things that was a benefit to young caregivers was online access to school and with that came more flexibility. Supports were also online, so there was better access to getting that when you needed it. Same with employment, a lot of folks’ employment shifted to virtual spaces and a lot of the youth who were looking for employment were looking for remote jobs which became more prevalent during the pandemic. So there was that, from a positive standpoint”* (online support group and outreach facilitator; volunteer; facilitated and scheduled support groups with adult and/or young caregivers and reached out to different organizations within the province to build awareness of and engage caregivers; had lived experience as a caregiver).

## 4. Discussion

The aim of this study was to emphasize the voices of service providers working with young caregiver clients during the pandemic, and to explore their perspectives on the impact of the pandemic on young caregivers and their families. As mentioned, this empirical research was part of a larger project that began in 2020 and remains ongoing until 2023. The study explored the impact of the COVID-19 pandemic on young caregivers aged 5–25 years and their families in Canada. Our overarching conceptual framework, the social determinants of health [30], informed the data analysis. The decision to include the perspectives of service providers in this research project was a concerted effort to achieve a more comprehensive understanding of the impact of the pandemic on young caregivers in Canada.

Service providers offer a unique perspective on the experiences of young caregivers and their families during the COVID-19 pandemic. The findings point to two primary overarching themes, namely (1) service providers’ responses to the pandemic and (2) observations by service providers about the impacts of the pandemic on young caregivers, and a secondary theme, (3) positive impacts of the COVID-19 pandemic on young caregivers, that emerged through the analysis.

This study illustrated the ways in which service providers attempted to address gaps in services to young caregiver clients and their families during the pandemic. The pandemic led to an influx in client demand for service providers supporting young caregiver clients. Specifically, service providers were required to adapt their service delivery methods in order to comply with public health guidelines and to meet the unprecedented demand for services. This facilitated changes in organizational priorities and the roles held by service providers prior to the pandemic. Service providers, in turn, shared how their work impacted their mental health as they struggled to maintain personal and professional boundaries while working from home during the pandemic. Importantly, service providers identified similar, simultaneous, and co-occurring impacts of the pandemic between their young caregiver clients and their families, including isolation, difficulties in navigating online spaces, and challenges in navigating boundaries while working from home with family members. Further, service providers shared experiences of seeking additional support and resources to manage their work demands during the pandemic, which paralleled the experiences of their clients.

This study highlights that young caregivers are not a homogenous group and that their experiences must be understood in a larger social and cultural context. Service providers identified the significant differences in the social locations and lived experiences of their young caregiver clients. The social determinants of health [30], as a conceptual framework, offer a more comprehensive understanding of the experiences of these young people and their families. For example, the findings of this study suggest that socioeconomic status and the availability of community resources were significant factors in the experiences of young caregiver clients and their families during the pandemic. Economic stress and a lack of resources (particularly internet access and internet-enabled devices such as laptops, tablets, and smartphones) contributed to increased referrals to service organizations and increased caseloads for service providers. Young caregivers and their families who resided in more remote communities experienced greater challenges in terms of access to the internet, which created challenges for young people participating in remote learning as well as remote programming offered by young caregiver organizations.

The research findings of this study support previous research in health contexts that has pointed to variations in the accessibility of healthcare services across the country, particularly highlighting the differences in northern and southern communities [36] and rural and urban communities [37]. Factors such as population density as well as demographic and socioeconomic characteristics are important factors in the availability of social and health services [36,37]. Findings suggested that regional variations were significant in the availability of services, support, and experiences of young caregiver clients and their families during the COVID-19 pandemic. For example, access to the internet and the availability of programs and services were significant barriers for young caregivers and their families in rural communities, similar to the findings of a previous study [10] that compared young caregivers who lived in rural versus urban communities in the Canadian context (pre-pandemic).

### 4.1. Strengths and Limitations

This study offers a unique insight into the experiences and voices of service providers working with young caregivers and their families during the COVID-19 pandemic. This knowledge helps to contextualize information provided by young caregivers and their families within the larger study. Further emphasizing the voices and experiences of service providers allows researchers and policymakers to better understand which resources have been effectively utilized and which areas may require further research and investment to best assist young caregivers and their families in Canada.

There are several limitations to this study. The first limitation is the small sample size of nineteen (19) service providers. This sample size is in no way representative of the entire population of service providers working with young caregivers and their families in Canada; therefore, the findings are not generalizable. This study represents a small population of service providers, from three (3) different organizations in Ontario, Canada. As Canada’s largest province, Ontario is the only province with established young caregiver organizations, thus drawing attention to the regional variations in service needs and delivery across the country. Social and health services fall under provincial and territorial jurisdiction in Canada; therefore, resource allocation and availability must be understood in this context.

### 4.2. Policy and Practice Implications

The findings of this study suggest several practice implications for service providers and organizations working with young caregivers and their families. Young caregivers and their families were uniquely impacted by the pandemic, which contributed to increased demands for services. Service providers described how sharing resources and knowledge between professionals was a significant asset to them as they navigated the ever-changing service landscape brought about by the pandemic. Additional support was required for service providers themselves, who were having to work from home in a potentially stressful environment, and with family members nearby, without the usual debriefing collegial support mechanisms, particularly in the context of burnout, grief, and loss.

There are significant implications for social policy in this area and there is a need to ensure that those most disadvantaged in society are not further disadvantaged by the pandemic—for example, due to a loss of income/support services or poorly developed or consumer-costly infrastructure needed for internet access. Service providers highlighted a critical need for increased funding for young caregivers and their families to reduce the financial impacts of the pandemic. Service providers suggested that additional financial resources for organizations and families would help to mitigate the impact of the pandemic and reduce the stressors experienced by young caregivers and their families.

## 5. Conclusions

This study offers a unique insight into the experiences and voices of service providers working with young caregivers and their families during the COVID-19 global pandemic. According to service providers, young caregiver clients and their families were considerably impacted by the pandemic. Subsequently, service providers working with young caregiver clients were challenged to respond to increased service demands and adapt their service delivery methods to ensure adherence to public health guidelines.

The voices of service providers emphasize the ways in which young caregiver clients represent an under-resourced population worthy of further investment and support. Aligning with the social determinants of health framework [30], investments in the wellbeing of young caregivers in Canada would ensure greater equity for these young people and their families. Socioeconomic status and the availability of local community resources were significant factors in mitigating the effects of the pandemic on young caregiver clients and their families. Service providers expressed the need for additional resources (primarily financial) for young caregivers in Canada.

Further research should explore the effectiveness of specific programs, services, and interventions from the perspective of service users. Additionally, it would be beneficial to re-evaluate the impact of the pandemic on young caregivers and their families from the perspective of service providers after public health restrictions were lifted nationally (and globally) as a point of comparison.

## Figures and Tables

**Table 1:** Roles of service providers.

Service Provider Role	Description of the Role
1. Clinical Support	Service providers described providing respite and support services to young caregivers, including offering safe spaces, reducing social isolation, normalizing caregivers’ feelings, and celebrating their successes. Additionally, service providers described teaching life skills and offering practical knowledge and guidance to young caregivers, including cooking, sewing, stress management, and financial literacy. Finally, service providers made referrals to clinical therapy programs and services for young caregiver clients.
2. Service Delivery and Facilitation	In the area of service delivery, service providers described their roles in socializing and connecting with young people, running programs (e.g., life skills), and facilitating online support groups in addition to website maintenance (since services were being offered remotely). Service providers offered opportunities for young people to socialize outside of their families and develop friendships with other young caregivers, in an effort to reduce isolation and facilitate breaks from caregiving.
3. Advocacy	A significant aspect of service providers’ work was to advocate for their young caregiver clients, specifically by highlighting young people’s voices and lived experiences within young caregiver organizations, with funders, and in the broader community. One participant described their advocacy work as “building awareness on the general community of the population through outreach at different regions across the province and connecting with potential organizations that would have an interest in the conversation” (online support group and outreach facilitator; volunteer; facilitated and scheduled support groups with adult and/or young caregivers and reached out to different organizations within the province to build awareness of and engage caregivers; had lived experience as a caregiver).

## Data Availability

Data sufficient for the reader to validate the article findings can be made available as appropriate upon request to the corresponding author.

## Supplementary Materials

The following supporting information can be downloaded at: https://www.mdpi.com/article/10.3390/ijerph20156446/s1, File S1: Semi-structured interview questions for directors/managers; File S2: Semi-structured interview questions for service providers who support young caregivers directly.
