# Supplementary material for: Voices from Service Providers Who Supported Young Caregivers throughout the COVID-19 Pandemic in the Canadian Context
Source: Int J Environ Res Public Health. 2023 Jul 26;20(15):6446. doi: 10.3390/ijerph20156446 (PMC10419008; doi:10.3390/ijerph20156446)
Supplement: Supplementary file 1 [file ijerph-20-06446-s001.zip › Interview Guide for Directors and Managers.pdf]

INTERVIEW GUIDE FOR DIRECTORS AND MANAGERS

Setting-up an Interview

1. Ensure participants (and their parents/legal guardians if applicable) have created a 6-digit code (and has a record of it) and filled out the correct online consent form by through searching of their 6-digit code on Google Forms **before** setting up a time to conduct the interview.
2. Clearly communicate that the interviews will occur via Zoom for Healthcare and in-person interviews are not permitted because of COVID-19 precautions.
3. Upon setting up a time for the interview:
    a. Advise participants (and their parents/legal guardians):
        i. They should have the interview in a quiet and private place that they feel safe sharing information
        ii. To contact Dr. Kristine Newman (Kristine.Newman@ryerson.ca) if they have any questions about the study
        iii. To contact NAME OF FACILITATOR (EMAIL OF FACILITATOR) if they wish to reschedule or cancel the interview
    b. Attach:
        i. The appropriate consent form(s)
        ii. The questions for the interview
4. Send participants a link for Zoom meeting (we will be using Zoom for Healthcare)

Conducting the Interview

1. Before starting the interview, you must review (aloud) the contents of the appropriate ***Information and Consent Form*** and address any questions as needed prior to starting the interview
2. Take fieldnotes to capture key findings that can help make future interviews better (i.e., confusing questions, questions not considered) or any challenges you or the participants experience during the interview

QUESTIONS

About Your Role and How COVID Changed It

1. Could you describe a typical workday in your role in 2019 (before the pandemic) and how that changed in 2020 (when the pandemic first started and public health restrictions were at their height)? Please mention if you have a director-level, managerial-level, and/or both roles and if you have/had different roles/titles since 2019.
2. Due to the diverse/developing/growing roles that a policy/decision-maker must fill in different sectors, it is often difficult to understand what policy/decision-makers sometimes do. As a policy/decision maker, what challenges did you face during

   2020 (when public health restrictions were being implemented and at their height)?
    1. What do you think your role is/will be during recovery (2021 onwards) from the pandemic and as restrictions lift?

The Program for Young Caregivers

3. Could you provide a brief description of the services your program for young caregivers offers?
    1. Could you describe the different roles/jobs each of your team members including which area(s) they serve?
    2. How many volunteers are on your young caregiver team? What roles to they play?
    3. How many paid staff are on your young caregiver team? What roles do they play?
4. Regarding your program for young caregivers, could you please talk about how you were able to identify the challenges young caregivers were facing?
    1. What were some of the key issues experienced by your young caregiver clients in 2020 (during the height of the pandemic)?
        1. Did this change over time (2021 onwards) and, if so, how did it change?
    2. What issues did you identify as priorities for your program to address with the resources you had available?
    3. What factors did you have to consider when making these changes (i.e., issues faced by clients, implementation challenges, financial)?
5. Did your program change as restrictions started to lift (2021 and 2022)? How did it change and what was your role in making this change? (i.e. program design, policy changes, staffing changes, additional staff training or hires to address the changing needs)
    1. What factors did you consider when making these changes (i.e., issues faced by clients, implementation challenges)?
    2. What plans do you have for the future of your program and/or how would you like to see it change/develop?

Researchers

6. What do you think researchers can do to empower/support/strengthen your personal capacity, your organization's capacity, and/or the system's capacity to support young caregivers? Feel free to mention COVID-specific and/or ongoing challenges/efforts/policies/issues and/or knowledge gaps/needs.

Politicians and Legislators

8. How do you think policy and decision-makers in government, such as politicians and legislators, could contribute to making it easier for you, your agency, and other institutions to support young caregivers at municipal, provincial, and national levels?

Funding Agencies

9. How do you think funders/sponsors could contribute to making it easier to support young caregivers in your sector and perhaps other sectors you are knowledgeable of and/or young caregivers engage with?
    1. Is there a particular funder(s), by name, that you would like to receive funding from? What are the COVID-specific and/or ongoing challenges to receive funding from this funder(s)?

Public

10. How do you think voters/Canadians in Ontario/other provinces/throughout Canada could help to improve the lives of and/or support efforts that empower young caregivers?

Other Stakeholders

11. Are there other stakeholders, institutions, and/or groups that you think could be and/or continue to be an asset in supporting young caregivers? For example, school boards, healthcare providers, scholarship funders, advertising agencies, YouTubers, influencers, etc
