# Supplementary material for: Voices from Service Providers Who Supported Young Caregivers throughout the COVID-19 Pandemic in the Canadian Context
Source: Int J Environ Res Public Health. 2023 Jul 26;20(15):6446. doi: 10.3390/ijerph20156446 (PMC10419008; doi:10.3390/ijerph20156446)
Supplement: Supplementary file 1 [file ijerph-20-06446-s001.zip › Interview Guide for Service Providers Working Directly with Young Caregivers.pdf]

GROUP INTERVIEW GUIDE FOR SERVICE PROVIDERS

Setting-up an Interview

1. Ensure participants have created a 6-digit code (and has a record of it) and filled out the correct online consent form by through searching of their 6-digit code on Google Forms **before** setting up a time to conduct the group interview.
2. Clearly communicate that the group interviews will occur via Zoom for Healthcare and in-person group interviews are not permitted because of COVID-19 precautions.
3. Upon setting up a time for the group interview:
    a. Advise participants:
        i. To find a quiet and private place that they feel safe sharing information with good phone reception or Wi-Fi access to join the group interview
        ii. To contact Dr. Kristine Newman (Kristine.Newman@ryerson.ca) if they have any questions about the study
        iii. To contact NAME OF FACILITATOR (EMAIL OF FACILITATOR) if you cannot make it to the group interview or are having difficulties joining the Zoom meeting
    b. Attach:
        i. The appropriate consent form(s)
        ii. The questions for the group interview
4. Send participants a link for Zoom meeting (we will be using Zoom for Healthcare)

Conducting the group interview

1. Before starting the group interview, you must review (aloud) the contents of the appropriate ***Information and Consent Form*** and address any questions as needed prior to starting the group interview
2. Take fieldnotes to capture key findings that can help make future group interviews better (i.e., confusing questions, questions not considered) or any challenges you or the participants experience during the group interview

Guiding Questions

1. Participant backgrounds:
    a. What geographical are you supporting (i.e., system, national, provincial, municipal, urban, rural) (2020 vs 2021)
    b. The nature of your roles and responsibilities during COVID (2019 vs 2020)
    c. What does your agency or organization do for your clients (2019 vs 2020)
2. Young caregivers' experiences: During 2020, what challenges/changes did you observe/hear about the pandemic causing for the young caregivers and their families? (i.e., did certain services change or disappear)
    a. How do you believe they navigated these challenges/changes? (i.e., from your organization [what was the feedback about the supports you provide] what supports did they receive; were those supports adequate)

    b. Did this change in 2021 (challenges and adaptations) and what do you think caused this change to occur? How did things get better, worse, or stay the same?

3. Looking at what your role was like before the pandemic, during 2020, and now (2021):
    a. In 2020, did more people access your services and why do you think this happened? What was the demand like for your services in 2020 vs now (2021)?
    b. In 2020, how did COVID impact/change the nature of the work/programs/services you provided? Is this still the same now (2021)?
    c. In 2020, what challenges did you face in providing services? (i.e.,) How are those challenges different or the same now (2021)?
    d. In 2020, what did you prioritize when supporting young caregivers and/or their families before the pandemic, in 2020, versus now (2021)? (i.e., educating young caregivers about the pandemic, provide fun activities to de-stress, counseling to discuss anxieties/uncertainties about the pandemic, coping with loss of "normal," friends, etc)
    e. What sources/resources did you use in 2020 and now (2021)? (i.e., Google, public health guidelines, social media)
        i. To keep up to develop programs?
        ii. To keep up to date with the latest information?
4. Looking back and thinking ahead:
    a. In 2020 and now (2021), are there any positives about the changes you had to make as a result of ongoing public health restrictions (you can talk about positives in your own role and for those you support)? (the pandemic has been a terrible thing, but some people have reported some positive experiences in their lives as a result of restrictions/changes such as being able to spend more time with love ones)
    b. In 2020, what would have made it easier to do your job well (i.e., guidelines, resources, awareness, internet access)? What about now (2021)?
    c. How do you think your role and services will change in the future as we navigate what seems to be an ongoing pandemic and new "normal?"
5. Anything else you would like to talk about or add that you think would help us make young caregivers' and their families' lives better during COVID?
